# Posterior Reversible Encephalopathy Syndrome (PRES) Associated With Eclampsia in a Pregnant Adolescent: A Case Report

**DOI:** 10.7759/cureus.114255

**Published:** 2026-08-10

**Authors:** Karla Alejandra Gómez, Yuli Estefania Rojas Cuadros, Gabriel Sierra Rosales

**Affiliations:** 1 General Practice, Universidad de Pamplona, Clínica Medical Duarte, Cúcuta, COL; 2 Pediatrics, Universidad Industrial de Santander, Clínica Medical Duarte, Cúcuta, COL; 3 Pediatric Neurology, Universidad Militar Nueva Granada, Clínica Medical Duarte, Cúcuta, COL

**Keywords:** brain magnetic resonance, posterior reversible encephalopathy syndrome (pres), postpartum eclampsia, severe preeclampsia, vasogenic brain edema

## Abstract

We report the case of a 16-year-old pregnant woman at 35.4 weeks’ gestation who was admitted with severe headache, lower extremity edema, and severe hypertension. Initial evaluation revealed significant proteinuria and elevated lactate dehydrogenase (LDH) levels, consistent with severe preeclampsia, leading to an emergency cesarean section. In the immediate postoperative period, the patient developed a generalized tonic-clonic seizure associated with refractory hypertension, requiring intravenous antihypertensive and antiseizure therapy.

Brain magnetic resonance imaging (MRI) demonstrated findings consistent with posterior reversible encephalopathy syndrome (PRES). Comprehensive management was initiated, including magnesium sulfate, strict blood pressure control, and anticonvulsants, resulting in a favorable neurological outcome without residual focal deficits. This case highlights the importance of early recognition of PRES in patients with preeclampsia/eclampsia, given its potential reversibility with timely treatment.

## Introduction

Posterior reversible encephalopathy syndrome (PRES) is an acute neurological condition characterized by headache, seizures, altered mental status, and visual symptoms, associated with typical findings on brain MRI [1]. It is a clinical-radiological entity first described in 1996, previously termed reversible posterior leukoencephalopathy syndrome (RPLS); its importance lies in its potential reversibility when diagnosis and treatment are timely [2]. PRES has been linked to various systemic conditions, particularly severe arterial hypertension, renal disorders, and states of endothelial dysfunction such as preeclampsia/eclampsia [3]. The reported incidence of PRES varies considerably according to the population studied and the underlying clinical condition. In obstetric patients with eclampsia, PRES is frequently identified on neuroimaging; however, its incidence in the general population remains uncertain [4].

We report the case of an adolescent who developed postpartum eclampsia complicated by PRES despite magnesium sulfate prophylaxis, with refractory hypertension and neuroimaging findings suggestive of both vasogenic edema and a possible cytotoxic component. This case highlights several uncommon clinical features and underscores the importance of early recognition of atypical presentations to optimize neurological outcomes.

## Case presentation

A 16-year-old pregnant woman at 35.4 weeks of gestation (based on first-trimester ultrasound) was admitted to the emergency department. At admission, the patient had a blood pressure of 138/97 mmHg, marked proteinuria, headache, elevated lactate dehydrogenase (LDH) levels, fetal growth restriction, and suspected placental abruption (Table 1). The clinical and obstetric findings prompted management as preeclampsia with severe features.

**Table 1:** Admission laboratory results

Laboratory test	Result	Reference range
White blood cell count	10.780 ×10^3^/µL	4.500-11.000 ×10^3^/µL
Neutrophils	64%	Up to 75%
Lymphocytes	21%	Up to 50%
Hemoglobin	10.2 g/dL	12-16 g/dL
Platelet count	281 ×10^3^/µL	150-450 ×10^3^/µL
Total bilirubin	0.23 mg/dL	0.3-1.2 mg/dL
Direct bilirubin	0.09 mg/dL	0.1-0.3 mg/dL
Indirect bilirubin	0.14 mg/dL	0.1-1.0 mg/dL
Alanine aminotransferase (ALT)	24 U/L	10-34 U/L
Aspartate aminotransferase (AST)	17 U/L	10-49 U/L
Serum creatinine	0.73 mg/dL	0.5-1.0 mg/dL
Prothrombin time (PT)	9.2 seconds	9.9-11.8 seconds
Partial thromboplastin time (PTT)	31 seconds	22-38 seconds
International normalized ratio (INR)	0.84	0.8-1.2
Lactate dehydrogenase (LDH)	303 U/L	85-230 U/L
Urine protein (spot sample)	100 mg/dL	1-14 mg/dL
Urine creatinine (spot sample)	129 mg/dL	29-226 mg/dL
Urine protein-to-creatinine ratio	0.78	<0.3

During the surgical procedure, a large retroplacental hematoma was observed, along with 40% placental abruption. The uterine cavity was otherwise unremarkable, and the amniotic fluid was clear. She presented with uterine atony refractory to uterine massage and oxytocin; consequently, a B-Lynch hemostatic suture was performed. The newborn showed adequate neonatal adaptation and was transferred to basic neonatal care. Subsequently, the patient was transferred to the Pediatric Intensive Care Unit for postoperative monitoring.

Seventeen hours after the cesarean delivery, the patient developed a generalized tonic-clonic seizure that resolved following the administration of midazolam. The event was considered consistent with eclampsia. Repeat laboratory tests revealed no significant abnormalities in hepatic or renal function. A non-contrast head CT scan demonstrated bilateral frontal, parietal, and occipital cortico-subcortical hypodensities, findings highly suggestive of PRES.

**Figure 1:**
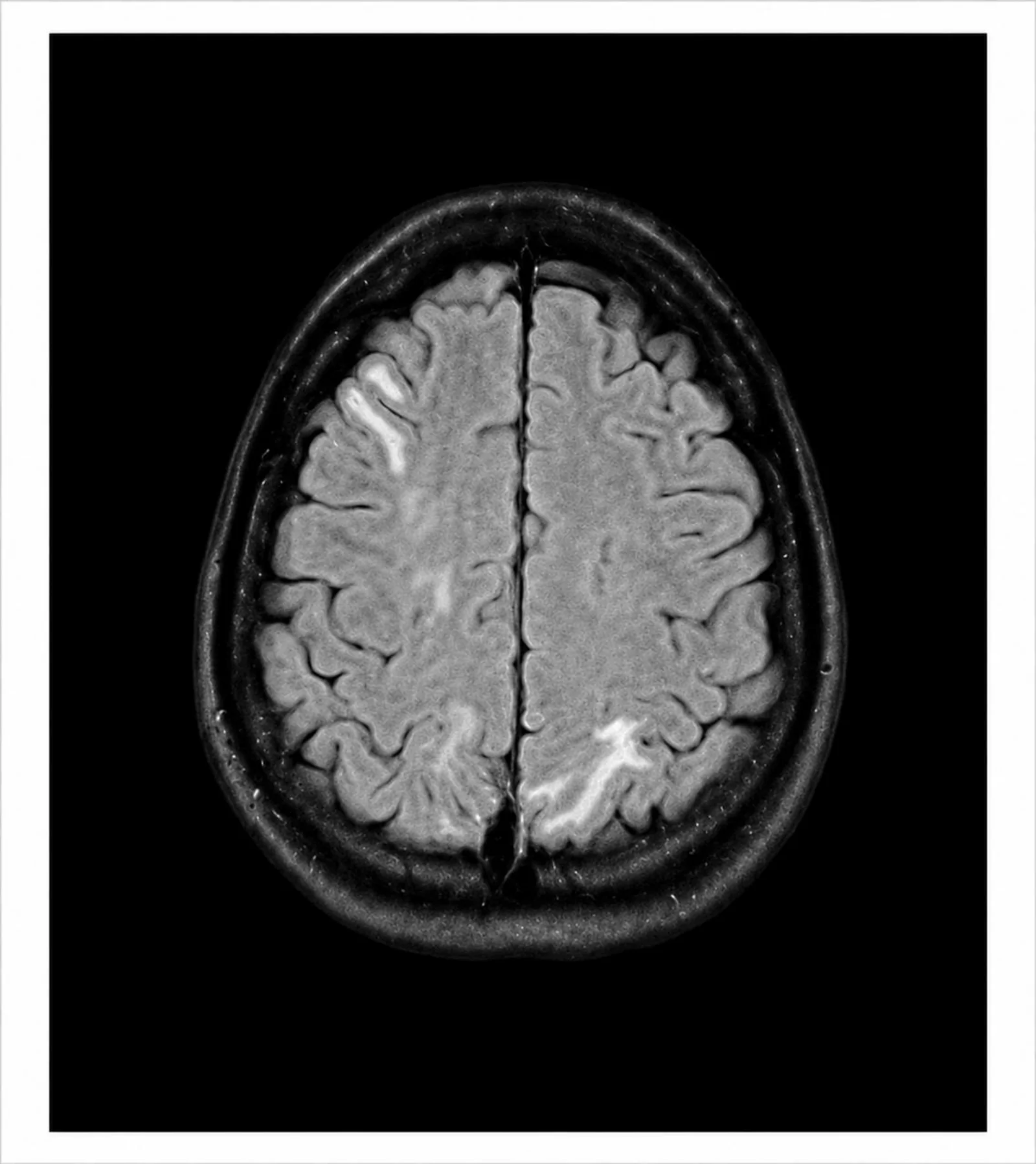
Brain magnetic resonance imaging (MRI) Axial T2-weighted MRI demonstrates bilateral asymmetric cortical and subcortical hyperintense signal abnormalities involving the parieto-occipital regions, consistent with vasogenic edema characteristic of posterior reversible encephalopathy syndrome (PRES).

During her intensive care unit stay, the patient continued to have refractory hypertension, requiring treatment with intravenous sodium nitroprusside and prazosin. Levetiracetam was also initiated for seizure prophylaxis. Brain MRI demonstrated bilateral parietal and occipital cortico-subcortical signal abnormalities, characterized by vasogenic edema (Figure 1), findings consistent with PRES.

The patient was evaluated by a pediatric neurologist, who confirmed the diagnosis and recommended continuing levetiracetam for one month, followed by an outpatient electroencephalogram (EEG) and subsequent gradual tapering of the antiseizure medication during follow-up. Her neurological status improved steadily, with only mild residual headache that responded to analgesic therapy and no recurrence of seizures. During hospitalization, she developed right lower lobe pneumonia, which was successfully treated with intravenous antibiotic therapy.

## Discussion

PRES is a neurological syndrome characterized by clinical findings like headache, altered mental status, seizures, and visual disturbances, associated with neuroimaging findings of vasogenic edema [5]. PRES has been strongly associated with acute hypertensive states, particularly eclampsia, which represents one of its leading causes in the obstetric population [6]. The pathophysiology is not fully established; however, two main mechanisms have been proposed. The first suggests that a sudden and severe elevation in blood pressure exceeds the limits of cerebral autoregulation, resulting in hyperperfusion, disruption of the blood-brain barrier, and subsequent vasogenic edema. The second mechanism involves endothelial dysfunction secondary to endogenous or exogenous toxins as observed in preeclampsia/eclampsia, sepsis, or exposure to immunosuppressive and cytotoxic agents. This endothelial injury increases vascular permeability, promoting fluid extravasation into the cerebral parenchyma. Together, these mechanisms account for the characteristic bilateral vasogenic edema seen on neuroimaging, predominantly affecting the parieto-occipital regions [7].

In this case, a pregnant teenager diagnosed with preeclampsia with severe features developed a generalized tonic-clonic seizure during the early postoperative period, presenting as postpartum eclampsia complicated by PRES. This point is clinically relevant, as eclampsia can manifest even after the termination of pregnancy [8] and despite magnesium sulfate prophylaxis, reflecting the persistence of systemic endothelial dysfunction beyond the immediate obstetric event.

In addition to significant proteinuria, the patient demonstrated elevated LDH levels, an indirect marker of tissue injury and endothelial activation [9]. These findings suggest an ongoing systemic inflammatory state that likely contributed to disruption of the blood-brain barrier [10]. The occurrence of seizures in the setting of refractory hypertension further supports failure of cerebral autoregulatory mechanisms, which were likely already impaired by the endothelial vasculopathy characteristic of severe preeclampsia [11].

A particularly relevant aspect of this case is that the neurological deterioration occurred during the immediate postpartum period, a stage in which significant hemodynamic alterations may persist, including abrupt changes in intravascular volume, fluid redistribution, and blood pressure fluctuations [12]. This physiological environment may serve as an additional trigger in patients with preexisting endothelial injury, facilitating the transition from severe hypertension to a well-defined neurological syndrome such as PRES.

Brain MRI is the imaging modality of choice for evaluating PRES in patients with preeclampsia/eclampsia, because it enables the identification of predominantly vasogenic cerebral edema [4-6]. On conventional sequences, lesions typically appear as hypointense or isointense areas on T1-weighted images and as hyperintensities on T2-weighted and fluid-attenuated inversion recovery (FLAIR) sequences, showing a bilateral distribution with a predilection for the parieto-occipital regions [4]. This predilection has been linked to the vulnerability of the posterior circulation territory to severe hypertensive crises and endothelial dysfunction; these factors promote impaired cerebral autoregulation, blood-brain barrier disruption, and fluid extravasation into the parenchyma, thereby explaining the typical neuroimaging pattern [5].

FLAIR sequences are particularly sensitive for detecting these lesions, which may extend to atypical regions such as the frontal and temporal lobes, insula, basal ganglia, thalamus, cerebellum, brainstem, and corpus callosum. Additionally, diffusion-weighted imaging (DWI) helps characterize the type of edema. In most cases, DWI findings are consistent with vasogenic edema; however, areas of restricted diffusion indicative of cytotoxic edema may occasionally be present and have been associated with more severe disease and an increased risk of irreversible neurological injury [4].

Regarding the imaging findings in this case, the initial CT scan revealed bilateral cortico-subcortical hypodensities, while MRI confirmed T2 hyperintensities in the parietal and occipital regions - a classic pattern of posterior vasogenic edema. Restricted diffusion may indicate a cytotoxic component and has been associated with a higher risk of irreversible injury; therefore, it supports close neurological monitoring and prompt control of the underlying hypertensive disorder [13].

The need for sodium nitroprusside to control refractory blood pressure levels highlights the extent of the vascular involvement. This suggests that, in this patient, the hemodynamic component played a decisive role in the pathophysiology of the condition, likely compounding pre-existing endothelial dysfunction. The combination of magnesium sulfate, intravenous antihypertensives, and anticonvulsants achieved clinical stabilization without neurological sequelae, confirming the syndrome's potentially reversible nature when timely treatment is administered.

Although specific evidence regarding PRES in adolescents is limited, this group is known to be more vulnerable to hypertensive disorders of pregnancy [14], possibly because of vascular immaturity and reduced hemodynamic adaptation. This case is therefore of particular clinical interest, as it illustrates the rapid progression of PRES in a young patient despite appropriate prophylactic management.

## Conclusions

PRES should be considered in patients with severe preeclampsia or eclampsia who develop seizures, severe headache, visual disturbances, altered mental status, or other neurological symptoms, including during the immediate postpartum period. This case illustrates that PRES may occur in the setting of postpartum eclampsia despite magnesium sulfate prophylaxis and highlights the importance of maintaining clinical suspicion in patients with persistent hypertension and neurological manifestations. The diagnosis of PRES was established based on the combination of clinical presentation and characteristic neuroimaging findings. Brain MRI remains the imaging modality of choice for supporting the diagnosis and distinguishing PRES from other acute neurological conditions. Early recognition and multidisciplinary management, including blood pressure control, magnesium sulfate administration, and antiseizure therapy when indicated, are essential components of care.

This case contributes to the limited published experience of PRES associated with adolescent pregnancy and highlights the need for further studies to better characterize its clinical presentation and outcomes in this population. The absence of follow-up MRI in this case represents a limitation, as radiological resolution would provide stronger confirmation of the reversible nature of the syndrome.
